# Predictors of Practice Patterns for Lymphedema Care Among Oncology Advanced Practice Nurses

**DOI:** 10.6004/jadpro.2012.3.5.4

**Published:** 2012-09-01

**Authors:** Joanne C. Ryan, Charles M. Cleland, Mei R. Fu

**Affiliations:** From New York University College of Nursing and College of Dentistry, New York, New York

## Abstract

Lymphedema, a debilitating and chronic condition, is considered to be one of the most distressing adverse effects of cancer treatment. The purpose of this study was to understand the practice patterns in lymphedema care and identify predictors influencing those patterns among oncology nurses, with a focus on advanced practice nurses. Random and purposive sampling was utilized to recruit 238 oncology nurses who completed the Web-based study. Participants included advanced practice nurses (nurse practitioners and clinical nurse specialists), nurse navigators/case managers, staff nurses, and directors/managers/coordinators. Data focused on perceived knowledge of and perceived competence in risk reduction, treatment, and self-management of lymphedema and practice patterns in lymphedema care. Actual knowledge of lymphedema care was evaluated. Descriptive, comparative, and regression analyses were performed. The study showed that perceived knowledge and perceived competence were highly correlated. Perceived competence was a predictor of practicing lymphedema care. Advanced practice nurses scored in the midrange for perceived knowledge and perceived competence in risk reduction and self-management, but obtained lower scores in perceived knowledge and perceived competence for treatment. The odds of advanced practice nurses delivering lymphedema care were less than those of staff nurses. This study identifies gaps and opportunities for advanced practice nurses to play an important role in providing lymphedema care, an essential aspect of cancer survivorship.

Cancer-related lymphedema is a chronic, debilitating condition often resulting from surgical removal of tumor, lymphadenectomy, or radiation therapy. The incidence of cancer-related lymphedema ranges from less than 1% to 48% depending on the cancer location and treatment as well as the definition and measurement of lymphedema (Cormier et al., 2010; Rockson & Rivera, 2008). Up to 40% of 2.5 million breast cancer survivors have developed lymphedema; in addition, it affects a large proportion of cancer survivors with other types of malignancies, including melanoma (16%), gynecologic (20%), genitourinary (10%), and head and neck (4%) cancer (Cormier et al., 2010). The impact of lymphedema stretches far and wide, affecting both the individual and the health-care system via physical (e.g., decreased limb function), psychological (e.g., mood, quality of life), and economic pathways (e.g., health outcomes, increased health-care costs, overall burden on the health-care system, etc.).

The Oncology Nursing Society (ONS) has developed professional competencies for advanced practice nurses (APNs) specifically for the oncology nurse practitioner (NP; ONS, 2007) and the oncology clinical nurse specialist (CNS; ONS, 2008). Competencies include responsibilities in the various aspects of cancer care, such as prevention, diagnosis, intervention, rehabilitation, and survivorship. Further commonalities across positions include the identification of cancer treatment-related risks, development and implementation of treatment plans, and patient and staff education. Likewise, lymphedema care encompasses risk reduction, prevention, treatment, and management of this chronic condition.

Consistent with the scope of responsibilities, lymphedema care should be an important tenet of the services provided by oncology APNs. Activities such as identifying lymphedema risks and symptoms, prescribing appropriate treatments, and referring to specialists (i.e., lymphedema therapists) are important aspects of an APN’s responsibilities. However, it is unknown whether APNs consider lymphedema care to be part of their role and responsibility, and little is known about the practice patterns of APNs as they relate to lymphedema care. The purpose of this study was to understand the practice patterns in lymphedema care and identify predictors influencing those patterns among oncology nurses, with a focus on APNs.

## Background

Lymphedema, which is an accumulation of protein-rich fluid in the interstitial space, can result from cancer and its associated treatment (Jensen, Simonsen, Karlsmark, & Bulow, 2010). As a chronic condition, lymphedema can have a direct impact on many facets of a patient’s life, including physical, functional, and psychosocial aspects (Fu & Rosedale, 2009; Paskett, Naughton, McCoy, Case, & Abbott, 2007). To date, there is no cure for lymphedema. Risk reduction, treatment, and self-management are important strategies to prevent lymphedema and minimize its negative impact on cancer survivors.

There is no period of time after cancer treatment when a patient is deemed to be safe from developing lymphedema (Ridner, Dietrich, & Kidd, 2011). The disorder results in significant physical changes that can impact body image, ability to perform activities of daily living (ADL), job function, and socialization/interpersonal relationships (Fu, 2005; Fu & Rosedale, 2009; Ridner, Dietrich, & Kidd, 2011). As this is a progressive condition, early lymphedema identification and diagnosis is key; yet at the same time, it can be challenging for clinicians because observable signs are often absent when lymphedema first develops (Cormier et al., 2010; Fu & Rosedale, 2009; Radina & Fu, 2011).

Although lymphedema can be a resultant complication of therapeutic interventions for cancer such as surgery or radiation, there are non–treatment-related risk factors for lymphedema as well, including age, weight, venous impairment, infection, and others (Cormier et al., 2010). Lymphedema treatment is intended to slow down the progression of disease and provide symptom relief. Standard of care treatment for lymphedema is divided into two phases: (1) clinician management at onset of an acute exacerbation and (2) self-management by the individual with lymphedema (Ridner, Dietrich, & Kidd, 2011).

The clinician management phase is accomplished through decongestive physiotherapy, exercise, surgery, or pneumatic compression therapy (Radina & Fu, 2011). These activities often require the assistance of health-care professionals trained in lymphedema management, thus contributing to the costs associated with this debilitating disorder.

Lymphedema self-management, which requires patients to incorporate new activities and make changes in lifestyle, is considered a lifelong process (Fu, 2005). Performing self-administered manual lymph drainage exercises specific for lymphedema patients, wearing and caring for compression garments, practicing thorough skin care, avoiding heavy lifting, and self-monitoring are among the daily self-management activities to which patients must adhere in order to prevent progression of lymphedema (Fu, 2005; Ridner, Dietrich, & Kidd, 2011). These activities are time consuming, costly, and distressing (Fu, 2005). However, lack of adherence to therapy and self-management may readily permit progression to more advanced lymphedema, where the signs and symptoms become irreversible.

Early detection, intervention, and adequate self-care can aid in preventing the progression of lymphedema and thus positively impact factors associated with the later stages such as increased health-care costs and worsening impact on patient quality of life (Ridner, Dietrich, & Kidd, 2011). Advanced practice nurses are in the perfect position to contribute to lymphedema diagnosis and treatment. Targeted assessment can facilitate early identification and diagnosis through activities such as asking specific questions regarding lymphedema risks and symptoms while taking a medical history and performing physical examinations that include the measurement of potentially affected areas. Additionally, making referrals to health-care professionals such as certified lymphedema therapists and mental health practitioners for diagnosis, treatment, and psychosocial support is within the scope of practice for APNs.

In addition, explicit patient education focusing on the risks, prevention, treatment, and self-management of lymphedema is crucial to adequately preparing patients and setting expectations. It is important to educate the patient about lymphedema and risk reduction prior to cancer treatment so that the patient is aware of and able to report lymphedema signs and symptoms to health-care providers in a timely manner. Lymphedema education should also be carried out throughout the treatment and survivorship periods to serve as reinforcement that may ultimately contribute to adherence to risk reduction and self-management activities.

Patient awareness of lymphedema can lead to the performance of risk-reducing activities such as promoting lymph fluid drainage, avoiding trauma to the affected limb, wearing compression garments, and treating minor injuries (Radina & Fu, 2011). Fu, Axelrod, and Haber (2008) investigated the effect of providing lymphedema information on symptoms and risk-reduction behaviors in 136 breast cancer survivors. The authors found that there was a statistically significant difference between survivors who received lymphedema information (53%) and survivors who did not (47%) in terms of lymphedema symptoms, cognitive outcomes, and behavior outcomes. Survivors who did *not* receive information reported significantly more lymphedema symptoms such as heaviness, aching, stiffness, impaired shoulder mobility, numbness, and tightness/firmness than those who did receive the information (t = 3.03; *p* < .01). Those who received the information reported practicing more risk-reduction behaviors (t = 2.42; *p* = .01), such as promoting lymph drainage, avoiding blood drawing/injections/blood pressure readings in the affected limb, and utilizing compression garments while traveling by air. The authors also reported that the breast cancer survivors identified nurses as the second-most frequent resource for lymphedema information/education after pamphlets.

Furthermore, Fu, Chen, Haber, Guth, and Axelrod (2010) conducted a multiple regression on the same sample to examine the effect of providing information on lymphedema symptoms while accounting for treatment-related risk factors. The investigators concluded that providing information about lymphedema had a significant reverse effect on lymphedema symptoms (B = ?1.35; *p* < .01) and that providing information along with treatment-related risk factors accounted for 13% of the variance (R2 = 0.13).

Similarly, Ridner (2006) queried 149 breast cancer survivors with and without lymphedema about their pretreatment lymphedema education (risk of lymphedema development and risk-reduction strategies). Patients with lymphedema on average were 8 years (standard deviation, 7.8) posttreatment while those without lymphedema were 5 years (SD 4.6) posttreatment. Patients reported having received lymphedema information most often from surgeons and nurses prior to surgery. However, in contrast, when asked where the patients would obtain lymphedema information now (years after cancer treatment), the internet was the most frequently identified resource, followed by oncologists and lymphedema therapists, while nurses fell low on the resource list. Given the importance of lymphedema care in a cancer patient’s experience and patient-centered outcomes, opportunities for APNs to play an important role in lymphedema care should be addressed.

## Methods

A cross-sectional and correlational design was utilized to achieve the objectives of the study. Institutional review board approval was obtained from New York University.

**SAMPLING** 

The Lymphedema Management Special Interest Group of the ONS, in partnership with the ONS Research Team, conducted this Web-based study. Random and purposive sampling was utilized to target 2,000 ONS members, with an expected minimum response rate of 10%, to ensure at least 200 participants in the study, The targeted ONS members were those who may have the opportunity of providing lymphedema care during daily clinical practice, specifically ONS members from the following ONS Special Interest Groups: Lymphedema Management, Radiation Therapy, Breast, Advanced Nurse Practitioner, and Surgical Oncology. An invitation to participate in the study was successfully e-mailed to 2,510 ONS members. The survey was available for completion from June to July 2009. Completion of the survey served as the respondents’ consent to participate. To ensure anonymity of the participants, the ONS research staff sent the e-mail invitation to potential ONS members and removed any identifiable information from the completed survey. The investigators only received the deidentified information.

**INSTRUMENT** 

Under the leadership of the principal investigator (M. R. F.), a group of seven lymphedema experts developed the survey. The survey consisted of 27 items assessing nurses’ perceived knowledge and competence regarding lymphedema care as well as their practice patterns. It contained three sections: (1) practice patterns, perceived knowledge, and perceived competence; (2) demographic questions related to role, work setting, and years of nursing experience; and (3) an optional section testing actual lymphedema knowledge. To make certain that broad and detailed information was collected, the survey items were developed using multiple formats including Likert-like scales (0 indicating least and 5 indicating most), multiple choice, and short-answer questions. Fifteen of the items pertained to nurses’ perceived knowledge, competence, and practice patterns in lymphedema care. Content validity was ensured by 10 oncology nurses who were not participants in the study.

The participants were asked to rate their *perceived knowledge* of lymphedema risk reduction, lymphedema treatment and lymphedema self-management using a scale ranging from 0 (not at all knowledgeable) through 5 (most knowledgeable). The participants were also asked to rate their *perceived competence* in the same three areas of lymphedema care utilizing a scale from 0 (not at all competent) through 5 (most competent). Additional items asked how important it was for nurses to provide services related to lymphedema risk reduction, treatment, and self-management on a scale from 0 (not at all important) through 5 (most important). Further questions asked for information on current practice patterns of lymphedema care in the work setting. Demographic data were assessed utilizing 12 other questions eliciting information on age, gender, education, role, practice setting, and years in practice. It was estimated that it would take 10 minutes to complete the first two sections of the survey. Participants had the option of taking an additional 15 minutes to complete 20 test questions assessing actual knowledge regarding lymphedema and its treatment and management.

**DATA DOWNLOADING AND VERIFICATION** 

Raw data were downloaded by ONS information technology staff using Microsoft Excel files. The human-in-the-loop (HITL) method was used to verify data accuracy and ensure minimum data errors (Sollenberger, Willems, Della Rocco, Koros, & Truitt, 2005; Fu et al., 2010); HITL refers to the need for human intervention when dealing with electronic data (Zaidan & Callison-Burch, 2009; Fu et al., 2010). There are two basic steps in HITL. Step 1 is to *determine the most constant items reflecting the real number of respondents*. In this study, there were eight constant items identified. These included the respondent’s age, gender, highest nursing degree completed, primary specialty, years of nursing experience, years of oncology nursing experience, primary position, and the state/country in which the respondent currently practices. For each of these questions, only one true answer could exist. Therefore, the sum of the responses for each question should come to 100%. Step 2 is to *identify the number of duplicated responses*; there were no duplicates in the study.

**DATA ANALYSIS** 

Participants’ responses were assessed using descriptive, comparative, and regression analyses. Demographic information as well as responses to questions regarding nurses’ knowledge, competence, and practice patterns were summarized. Descriptive data were analyzed by the survey software. Additional analyses of the survey data were conducted in R software (R Development Core Team, 2011) and SPSS version 18. Chi-squared test, Fisher’s exact test, t-tests, Pearson’s correlations, and logistic regression models were utilized to analyze the data and generate comparisons and predictions. All statistical tests were two-sided with statistical significance achieved at *p* < .05, and all estimates were reported with 95% confidence interval (CI).

## Results

Of the 2,510 nurses who were invited to participate in the study, 532 opened the invitation. The survey was completed by 256 nurses for an overall response rate of 48%. The majority of the respondents reported being between 45 and 60 years of age (70%), were female (99.2%), had their highest level of nursing education at or below a bachelor’s degree (58.2%), worked primarily with adult patients (98.8%) in an outpatient setting (69.1%) in the United States (97.7%) (within 44 identified states), and had more than 20 years of oncology nursing experience (38.7%); see Table 1. Of the 256 nurses, 32 self-identified as NPs and 41 as CNSs, resulting in more than a quarter of the sample being APNs (27.5%). Other primary positions included academic educator, nurse navigator/case manager, clinical trials nurse, consultant, staff nurse, director/manager/coordinator, and other (Table 2). More than half of the nurses noted that their primary specialty was in medical oncology (171) while other specialty areas included blood/marrow transplantation (7), palliative care (15), prevention/detection (17), radiation oncology (13), surgical oncology (49), and other (57). It is important to note that some participants identified more than one specialty.

**Table 1:**
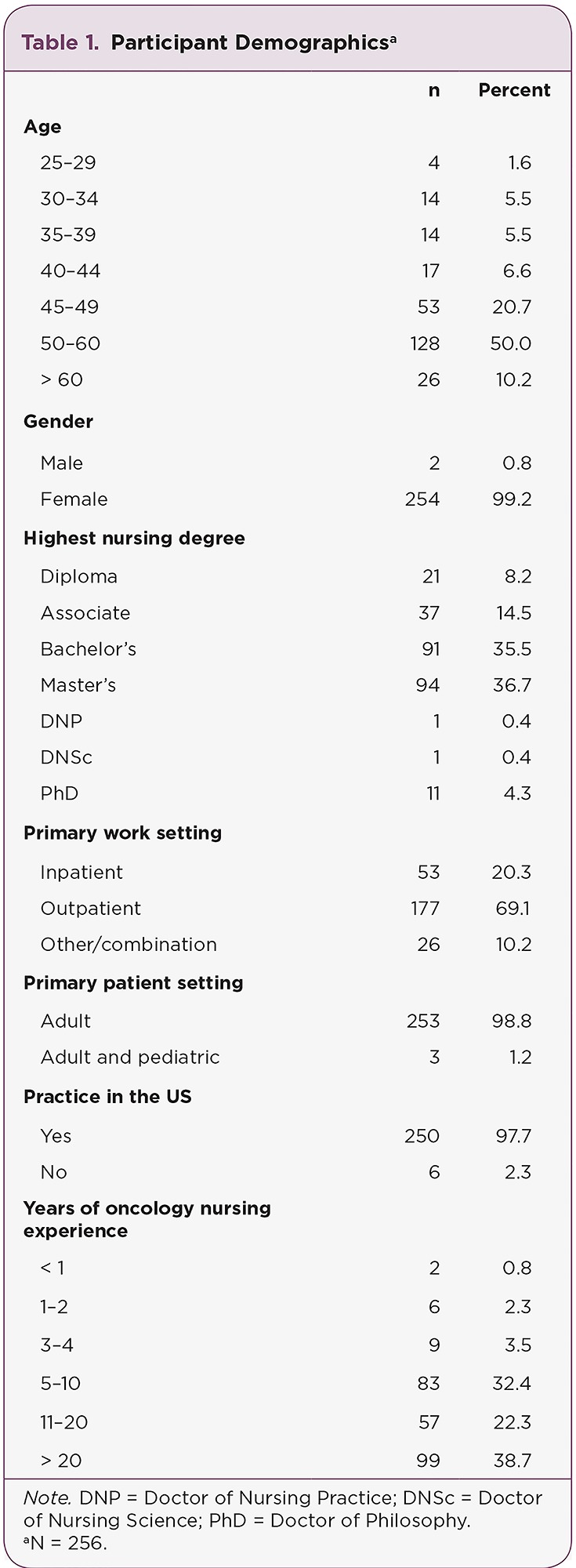
Table 1. Participant Demographics

**Table2:**
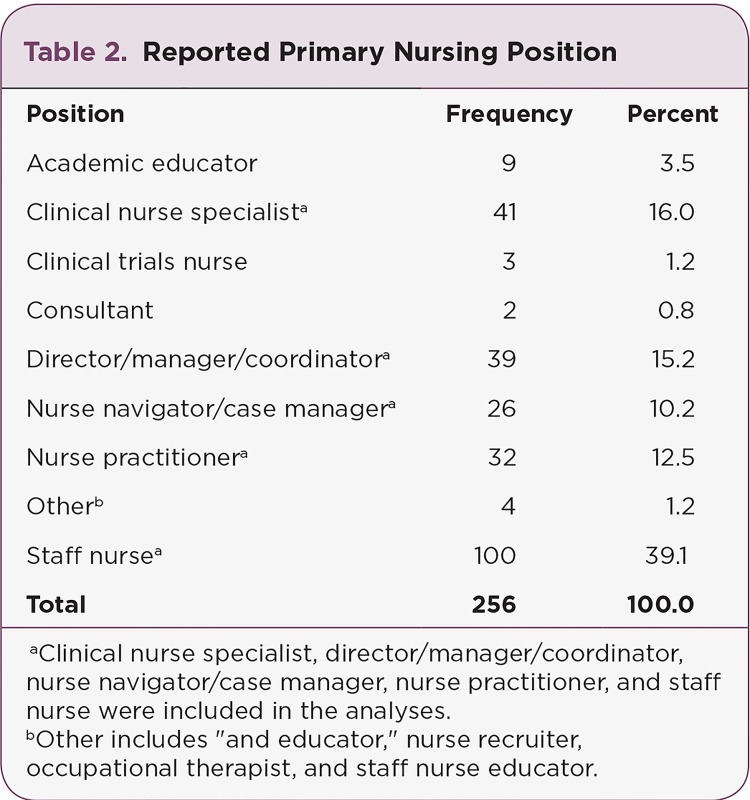
Table 2. Reported Primary Nursing Position

For the purposes of this article, the analyses were conducted on nurses active in clinical practice as this study focused on practice patterns in lymphedema care. Thus it was necessary that the participants’ responses reflected their actual clinical practice. The participants self-identified as NPs, CNSs, nurse navigators/case managers, staff nurses, and directors/managers/coordinators, bringing the sample population to 238 respondents. No significant differences in age were found across these five groups and, as expected, nurses with the more advanced positions had higher levels of education.

**PERCEIVED KNOWLEDGE AND COMPETENCE** 

**Figure 1:**
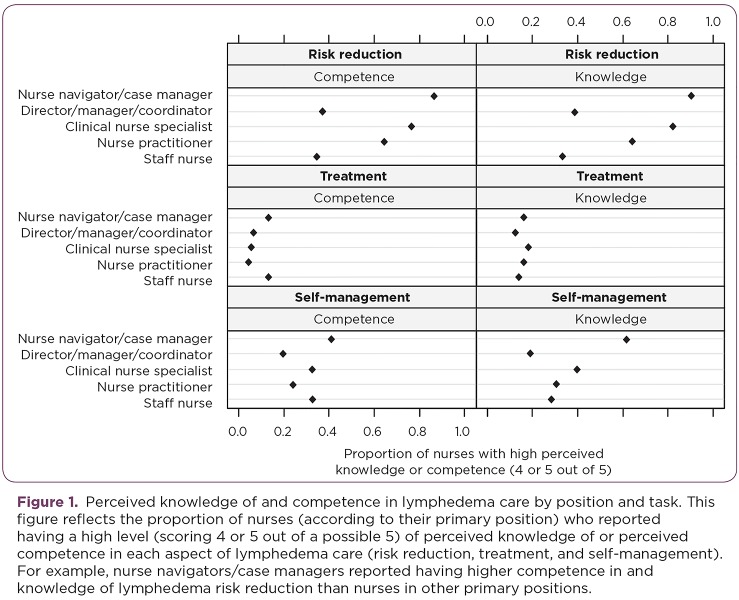
Figure 1. Perceived knowledge of and competence in lymphedema care by position and task. This figure reflects the proportion of nurses (according to their primary position) who reported having a high level (scoring 4 or 5 out of a possible 5) of perceived knowledge of or perceived competence in each aspect of lymphedema care (risk reduction, treatment, and self-management). For example, nurse navigators/case managers reported having higher competence in and knowledge of lymphedema risk reduction than nurses in other primary positions.

The perceived knowledge of and competence in lymphedema risk reduction, treatment, and self-management by primary nursing position are highlighted in Figure 1, where the ranking reflects the proportion of respondents with ratings of 4 or 5 on a scale of 0 to 5. According to primary position, perceived knowledge of and competence in *risk reduction* were highest for nurse navigators, followed by CNSs, NPs, directors/managers/coordinators, and then staff nurses. The scores for perceived knowledge of and competence in lymphedema *treatment* were low for all nursing position subgroups. Lastly, nurse navigators/case managers self-reported as having both the most knowledge of and competence in lymphedema self-management. CNSs ranked second in knowledge followed by NPs, staff nurses, and directors/managers/coordinators. Second in *self-management* competence were staff nurses, who ranked higher than CNSs, NPs, and directors/managers/coordinators.

Pearson’s product-moment correlation analyses were performed to determine the strength of the relationship between perceived knowledge and perceived competence for each of the lymphedema care areas. Overall, the results demonstrated that there was a strong relationship between the clinical nurses’ perceived knowledge and perceived competence within all three areas of lymphedema care. Perceived knowledge and competence were highly correlated for lymphedema risk reduction (r = 0.89, 95% CI = 0.85–0.90; *p* < .05 ), for lymphedema treatment (r = 0.75, 95% CI = 0.68–0.79; *p* = < .05), and for lymphedema self-management (r = 0.86, 95% CI = 0.82–0.88; *p* < .05).

**ASSESSMENT OF ACTUAL LYMPHEDEMA KNOWLEDGE** 

Twenty optional questions were posed to gain insight into the actual knowledge of nurses with respect to lymphedema and its treatment. All 238 nurses in this sample answered all 20 lymphedema knowledge questions.

The topics for the 20 optional questions and resulting numbers and percentages of correct scores by nursing position are shown in Table 3. The survey questions focused on the lymphatic system as well as lymphedema risk reduction, treatment, and measurement. The lowest percentage of correct scores was 14% for all nursing positions combined and was the result of the first question which asked about the general lymphatic system (this was also the lowest score across all questions for staff nurses at 17.0%, NPs at 6.3%, CNSs at 17.1%, directors/managers/coordinators at 10.3%, and nurse navigators/case managers at 19.2%). The highest percentage of correct scores was 88.7% for all nursing positions combined and reflected the answers to a question about patient education around lymphedema risk reduction (this was also the highest percentage of correct scores for staff nurses at 88.0%, NPs at 93.8%, directors/managers/coordinators at 87.2%, and nurse navigators/case managers at 96.2%). There were no statistically significant differences among the groups with the exception of question 15 ("Patients who are at risk for and have lymphedema should be instructed to report the following *except*:"), where the Pearson’s chi-square demonstrated a significant difference (÷2 = 9.892, *p* = .04). For this question, the NPs and nurse navigators/case managers were more likely than nurses in other positions to give a correct answer.

**Table 3:**
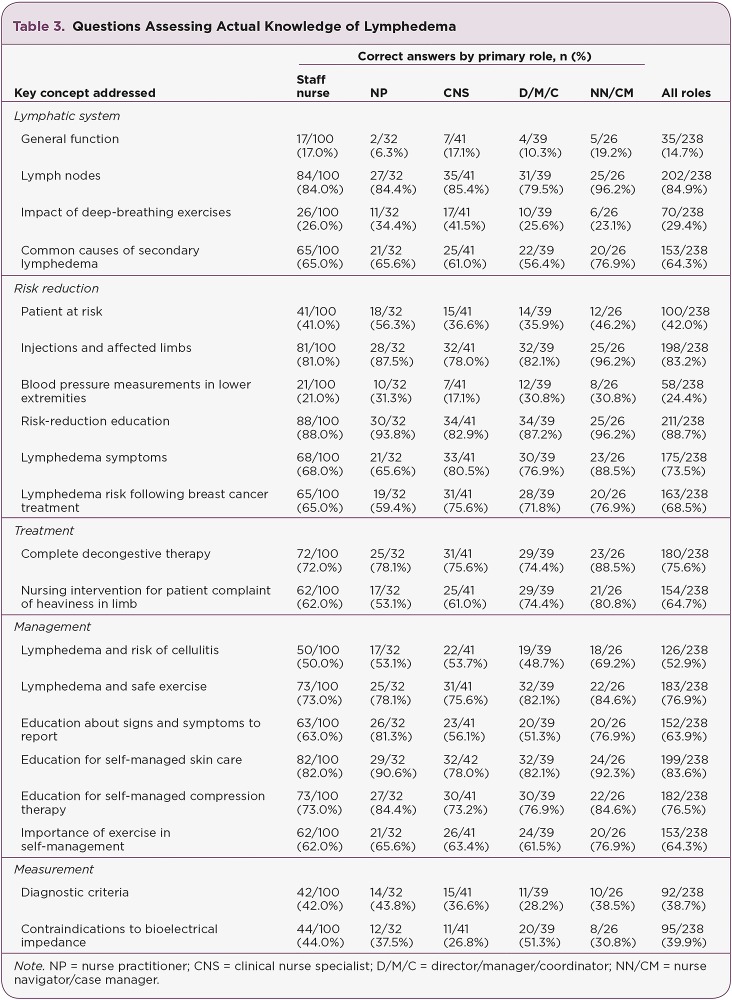
Table 3. Questions Assessing Actual Knowledge of Lymphedema

**Figure 2:**
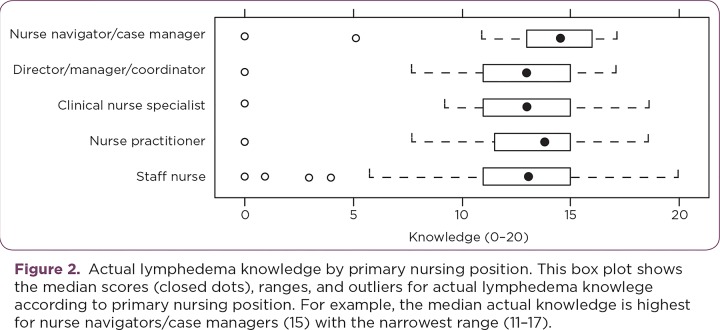
Figure 2. Actual lymphedema knowledge by primary nursing position. This box plot shows the median scores (closed dots), ranges, and outliers for actual lymphedema knowlege according to primary nursing position. For example, the median actual knowledge is highest for nurse navigators/case managers (15) with the narrowest range (11–17).

Knowledge scores (medians and ranges) by nursing position can be found in Figure 2. Nurse navigators/case managers demonstrated the highest knowledge and least variability, while staff nurses had the lowest scores and the most variability. The scores for NPs, CNSs, and directors/managers/coordinators were generally similar. Overall, the mean scores were not significantly different from each other (*p* = .24) and the means for the percentage of items answered correctly fell between 65% and 75%.

**PRACTICE PATTERNS AND PREDICTORS** 

**Figure 3:**
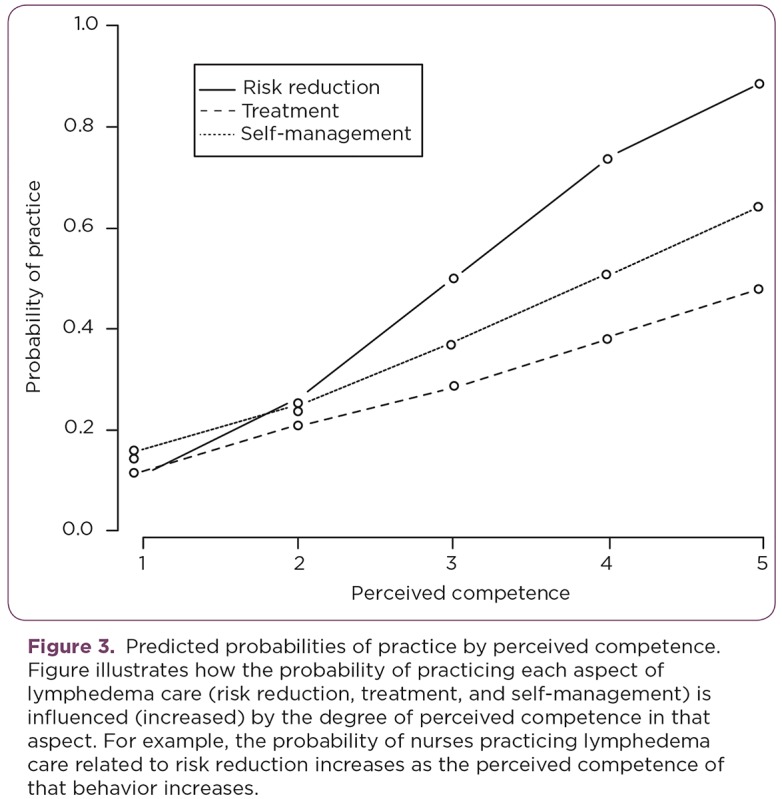
Figure 3. Predicted probabilities of practice by perceived competence. Figure illustrates how the probability of practicing each aspect of lymphedema care (risk reduction, treatment, and self-management) is influenced (increased) by the degree of perceived competence in that aspect. For example, the probability of nurses practicing lymphedema care related to risk reduction increases as the perceived competence of that behavior increases.

**Figure 4:**
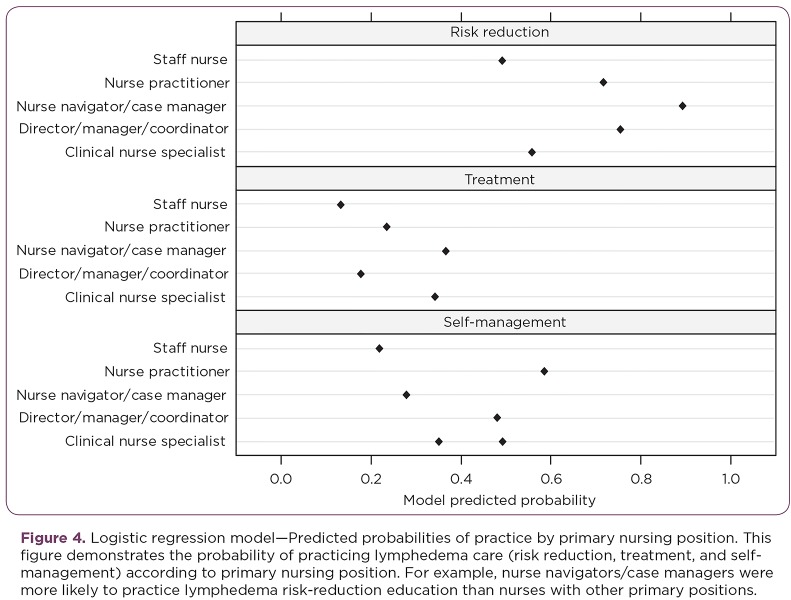
Figure 4. Logistic regression model—Predicted probabilities of practice by primary nursing position. This figure demonstrates the probability of practicing lymphedema care (risk reduction, treatment, and self-management) according to primary nursing position. For example, nurse navigators/case managers were more likely to practice lymphedema risk-reduction education than nurses with other primary positions.

A logistic regression analysis was conducted to identify potential predictors that may influence the practice patterns of nurses regarding lymphedema care. The results indicated that perceived competence in lymphedema risk reduction, treatment, and self-management could be considered predictors of the practice of educating patients about lymphedema (see Figure 3). Higher perceived competence in risk reduction significantly increased the odds of providing risk-reduction education (odds ratio [OR] 2.75, 95% CI = 2.1–3.72; *p* < .01). Similarly, higher perceived treatment competence significantly increased the odds of providing education about lymphedema treatment (OR 1.64, 95% CI = 1.25–2.17; *p* < .01). Likewise, higher perceived competence in self-management also significantly increased the odds of providing education regarding self-management (OR 1.8, 95% CI = 1.43–2.3; *p* < .01). Nurse navigators/case managers were the most likely to educate patients about lymphedema risk reduction and treatment, while NPs were more likely to educate patients on lymphedema self-management (Figure 4).

Participants were asked whether or not lymphedema care was considered a responsibility of nursing in their practice setting. When lymphedema care was considered a responsibility of nursing, the odds of providing such care increased for all three lymphedema activities (risk reduction: OR 8.46, 95% CI = 2.94–28.95; treatment: OR 2.52, 95% CI = 1.16–5.46; self-management: OR 2.73, 95% CI = 1.39–5.57).

Additional analyses were conducted investigating the aspects of lympedema care and comparing nurses according to primary nursing position (i.e., compared results for APNs [NPs and CNSs combined], directors/managers/coordinators, and nurse navigators/case managers to staff nurses). For risk reduction, the odds of providing education were increased for directors/managers/coordinators (OR 2.72, 95% CI = 1.05–7.48) and for nurse navigators/case managers (OR 7.65, 95% CI = 1.86–53.57) when compared to staff nurses. With regard to education about treatment, only nurse navigators/case managers showed an increase in the odds over staff nurses (OR 3.52, 95% CI = 1.22–10.07). Lastly, when compared to staff nurses, being a director/manager/coordinator increased the odds of providing education around lymphedema self-management (OR 2.34, 95% CI = 1.5–5.55). Surprisingly, being an APN did not increase the odds of providing education in any aspect of lymphedema care when compared to staff nurses.

## Discussion

Most nurses believed that risk reduction (95%) and self-management (68%) were the responsibility of nursing while 69% felt that treatment of lymphedema was the responsibility of a different discipline, such as lymphedema therapy. Similarly, participants answered that nurses should be reimbursed for education about risk reduction and self-management (79% and 67%, respectively) while only 37% felt that reimbursing for treatment was also warranted.

The literature and clinical practice experience suggest that nurses are a primary source for patient education, including information about lymphedema. The results of this study indicate that perceived knowledge of and perceived competence in lymphedema care are highly correlated and that these factors are predictors of nurses’ practice of lymphedema care. Advanced practice nurses scored in the midrange of nurse subgroups for perceived knowledge and competence in lymphedema risk reduction and self-management and scored low in perceived knowledge and competence for lymphedema treatment. While perceived knowledge and competence were highly correlated in this study, it is likely that there are additional contributing factors to competence such as the ability to practice what has been learned. Lymphedema treatment is highly specialized care, and the majority (69%) of the participants perceived lymphedema care to be the responsibility of a discipline other than nursing. It is possible that while APNs perceive themselves as having knowledge regarding lymphedema treatment, they do not have the opportunity to engage in this practice and therefore reported lower perceived competence. Perceived competence in risk reduction had the highest odds ratio for predicting practice while competence in treatment had the lowest, yet it was still a statistically significant predictor. Given these findings, further investigation of factors contributing to perceived competence would be warranted in future research.

The majority of nurses indicated that risk reduction (95%) and self-management (68%) were responsibilities of nursing. When nurses perceived that lymphedema care was a responsibility of the nursing profession, the odds of practicing care increased. However, when examining the odds of APNs practicing lymphedema care compared to that of staff nurses, there was no increase for any aspect of care (risk reduction, treatment, or self-management). APNs, with their advanced training and skills in diagnostics and treatment, are capable of providing multiple levels of lymphedema-related care, including and beyond patient education. The APN competencies put forth by ONS are aligned with the risk reduction, treatment, and self-management aspects of lymphedema care. However, this survey indicates that APNs had lower odds of delivering lymphedema care compared to staff nurses.

Actual knowledge of lymphedema was low in this sample, with the percentage of correct answers to the 20 survey questions ranging from 14% to 88.7% (when the subgroups of nurses were combined). With one exception (education about reporting lymphedema signs and symptoms), there were no significant differences among the nursing position subgroups, suggesting that despite the scores for perceived knowledge, nurses’ actual knowledge of lymphedema needs to be improved. Nurse practitioners had a higher percentage of correct scores over the other nurse subgroups for questions related to education about risk-reduction behaviors, education about reporting signs and symptoms of lymphedema, and diagnostic criteria, while CNSs had a higher percentage of scores for only one question related to the effect of deep breathing exercises on the lymphatic system. As previously noted, information given to cancer patients can have a significant impact on their symptoms and and risk-reduction practices. The generally low knowledge of lymphedema by the nurses in this sample indicates a critical need for more provider education.

Limitations of this study include small numbers of nurses in some subgroups of practitioners (41 CNSs, 39 directors/managers/coordinators, 26 nurse navigators/case managers, 32 NPs), the fact that respondents represented most (44) but not all states in the United States, and the fact that the survey was sent only to nurses who are members of ONS. Therefore, this sample may not be reflective of the overall population of oncology nurses practicing in the United States. Additionally, the actual knowledge section was limited to 20 questions and therefore likely does not reflect all aspects of lymphedema care with which the nurses might be familiar, possibly affecting the resulting scores.

## Recommendations

The results of this study demonstrate the existence of a lymphedema-related knowledge gap among oncology APNs as well as nurses in other primary positions. Closing this gap may be accomplished through the introduction of lymphedema content into the curriculum in schools of nursing, in professional conferences, in journals, and in practice settings.

Resources on lymphedema care can currently be found in the literature, through the ONS, and via specialty organizations such as the National Lymphedema Network. The ONS has a Lymphedema Management Special Interest Group that consists of nurses with interest and expertise in lymphedema care. This group can serve as a resource for nurses interested in expanding their knowledge about lymphedema, and as suggested by this study, may result in an increase in providing lymphedema care to patients truly in need.

Establishing and working in a multidisciplinary team can readily facilitate knowledge sharing. Identifying a colleague with expertise in lymphedema can result in an opportunity to learn from this individual and to share critical knowledge with others in the workplace. Gaining an understanding of the role of a certified lymphedema therapist not only provides APNs with an additional resource for which to refer their patients but may also offer an opportunity for career development into this specialty.

In summary, the findings of this study acknowledge the existence of gaps and opportunities for the APN with respect to education and practice. Providing further opportunities to put new knowledge into action can positively contribute to APN competence and enhance the delivery of quality care to cancer patients at risk for or experiencing lymphedema.
